# Luminescent color control of Langmuir-Blodgett film by emission enhancement using a planar metal layer

**DOI:** 10.1038/s41598-018-35467-4

**Published:** 2018-11-20

**Authors:** Ryotaro Ozaki, Tatsuya Yamada, Shinji Yudate, Kazunori Kadowaki, Hisako Sato

**Affiliations:** 1 Department of Electrical and Electronic Engineering and Computer Science, Graduate School of Science and Engineering, Ehime University, Matsuyama, 790-8577 Japan; 2 Department of Chemistry, Graduate School of Science and Engineering, Ehime University, Matsuyama, 790-8577 Japan

## Abstract

A metal enhanced emission of more than 20-fold is observed from a Langmuir-Blodgett (LB) monolayer on a planar aluminum layer with a polymer spacer. The spectral change of the metal enhanced emission using the metal layer is discussed experimentally and theoretically. Finite-difference time-domain simulations and transfer matrix calculations have been performed to investigate the cause of the enhancement. The analytical solution of the enhancement factor of the interference enhancement is also derived assuming the planar aluminum layer as a perfect electric conductor. Furthermore, we have demonstrated control of emission color of the LB film from the yellow-green to blue or red using the metal enhanced emission.

## Introduction

Luminescent transition metal complexes have been applied as emitting elements for photo-responsive devices^1–13^. Among them, cyclometalated Ir(III) complexes are attracting extensive attention due to their high emission properties in the visible region. Due to their advanced optical properties, they are studied to improve emission efficiency of organic light emitting diodes or to develop new molecular imaging probes. We have also demonstrated that nanometer-thick films prepared by Langmuir-Blodgett (LB) method work as an oxygen sensor^14–17^. Their functions are based on the property that energy transfer takes place efficiently from the triplet excited state of an Ir(III) complex to semiconductors or an oxygen molecule in a triplet ground state^13,18^. Since the desired spectral range of emission wavelength depends on its application, various Ir(III) complexes have been explored for tuning emission maxima^1–16,18^. However, it is not easy to control the color while maintaining emission intensity through a chemical approach.

The color of a material can also be changed in a physical way using the nanotechnologies such as quantum dots, photonic crystals, and plasmonics. Quantum dots are tiny particles or nanocrystals of a semiconducting material several nanometers in size^19^. In the quantum dots, electrons are confined in a small space, and their optical and electronic properties differ from those in bulk. Therefore, the colors emitted by quantum dots are determined by their sizes and shapes^20,21^. Photonic crystals having a periodic structure with a periodicity equivalent to an optical wavelength have photonic band gaps in which the existence of a certain energy range of photons is forbidden^22,23^. The photonic crystals having a photonic band gap in visible region show structural colors because the band gap corresponds to the reflection of light. Not only the photonic crystals but also nano-structures such as thin films, multilayers, and gratings also exhibit structural colors depending on the refractive index, the angle of incidence, and the size of nanostructure^24–26^. The metal nanoparticles and metal nanostructures also exhibit unique properties based on the surface plasmon resonance^27–29^. The colors of plasmonic nanoparticles with different sizes show a variety of different colors because localized surface plasmon resonance depends on its size, shape and the surrounding medium. Thus these nanostructured materials allow us to create various colors by controlling their size and shape.

Fluorescence enhancement using metal nanoparticles or various metallic nanostructures have been studied to improve luminescence performance^30–38^. In particular, silver island films and nanoparticles have been well examined to increase fluorescence intensity and to elongate fluorescence lifetime^32–34^. Plasmon enhanced fluorescence can be observed from molecules located at a certain distance from plasmonic nanostructures because fluorophore emission quenching occurs in molecules near a metal surface. Plasmon enhanced fluorescence has been reported not only from the island films but also from evaporated metal layers^35,36^, in which the emission can be enhanced by using surface plasmon coupling with a thin metal layer having surface roughness and imperfection. A more than ten-fold enhancement of light emission was observed from the dye doped polymer on the thin silver layer. On the other hand, interference enhancement from a silver layer has also been demonstrated to improve luminescence efficiency^37,38^. The effects of planar metal layers on the fluorescence intensity and lifetime have also been extensively studied^37–44^. Although two research fields deal with very similar systems, one is interpreted as plasmon enhancement, while the other as interference enhancement. Regardless of mechanism or interpretation, most of the researches of the metal enhanced luminescence have been focused on the enhancement factor and lifetime. In this study, the spectral change of metal enhanced emission using a thin metal layer is mainly discussed experimentally and theoretically. We have experimentally observed a 20-fold enhancement from an Ir(III) complexes LB film on a planar aluminum layer. Fluorescence properties nearby a planar metal layer have been analyzed by using electric dipole model^37–44^, we here have calculated optical properties of the LB film on the planar aluminum layer by finite-difference time-domain (FDTD) simulations and transfer matrix calculations. A simple analytical solution of the enhancement factor of the interference enhancement is also derived assuming the planar aluminum layer as a perfect electric conductor. Furthermore, we have demonstrated control of emission color of the LB film from the yellow-green to blue or red using the metal enhanced emission.

## Results

**Figure 1:**
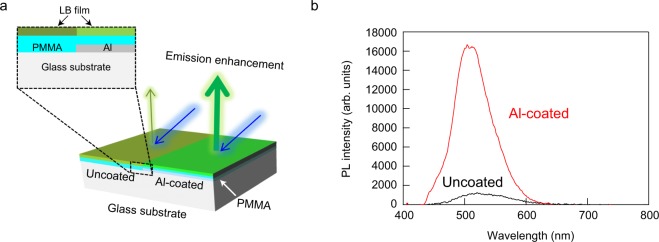
Experimental setup and emission spectra from the LB film on aluminum coated and uncoated regions. (**a**) Schematic experimental setup to measure the metal enhanced emission. (**b**) Photo luminescence (PL) spectra of the LB film on aluminum coated and uncoated regions.

The schematic illustration of the experiment to observe metal enhanced emission from an LB film is shown in Fig. 1a. Half of the substrate was coated with aluminum but the other half was uncoated for comparison. Poly(methyl methacrylate) (PMMA) was spin-coated on the aluminum surface to fabricate a spacer between the LB film and aluminum layer. Figure 1b shows the photo luminescence (PL) spectra from the LB monolayer excited by a 405 nm laser diode with p-polarization. The spacer and aluminum layer thicknesses in this sample were 218 and 20 nm, respectively, which were estimated from the transmission spectrum by curve fitting. It is clear that the PL emission from the LB film on the aluminum coated glass is greatly enhanced than that on the non-aluminum coated glass. The emission enhancement at 500 nm is 18-fold. This enhancement factor is relatively high compared with previous studies^33–36^.

**Figure 2:**
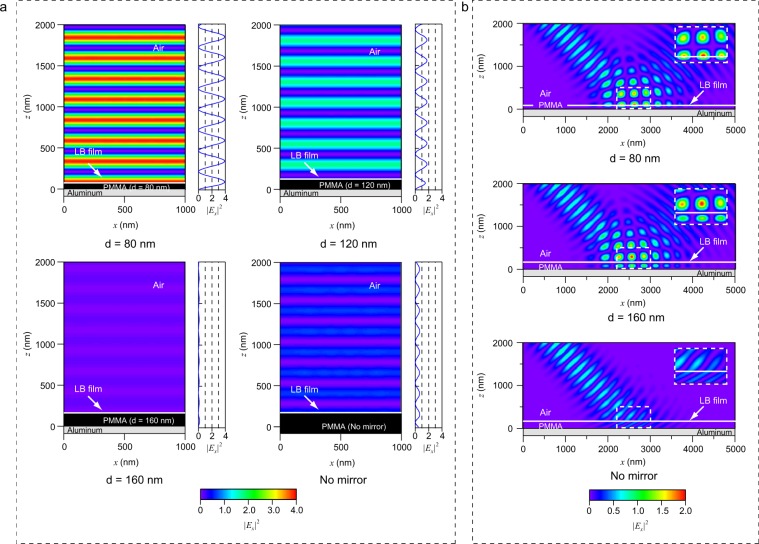
PMMA spacer thickness dependences of emission and excitation light intensities calculated by the FDTD method. (**a**) Electric field profiles of emission light from the LB films with a wavelength of 500 nm. (**b**) Electric field profiles of excitation light with a wavelength of 405 nm and p-polarization.

We have performed FDTD simulations to analyze whether the cause of the enhancement is either plasmon or interference. In the calculations, the metal surface was assumed as a flat and perfect electric conductor surface. Since the flat perfect conductor cannot excite plasmon, the calculated results show only the interference effect. Figure 2a shows the PMMA spacer thickness dependence of the light intensity emitted from the LB film. We assumed the LB film as a flat light emitter which is represented as a white line in Fig. 2a. The p-polarized light source with a wavelength of 500 nm and amplitude of 1 was set in the LB film layer. The emitted light from the LB film traveled upward and downward, and then the downward light was immediately reflected by the aluminum layer as a mirror. Therefore, the light intensity in the air results from the superposition between the upward light and the reflected light. As the result of interference, it is clear that the emission intensity depends on the PMMA thickness *d*. Here, the electric fields in the PMMA spacer are not shown in the 2D plots to focus on the electric field profiles in the air. Note that the interference light intensity for *d* = 80 nm is approximately four times of the light source. This is because the amplitude of electric field will be twice in constructive interference. In this condition, the intensity becomes four times larger than that of the original wave. In contrast, the light intensity for *d* = 160 nm is approximately zero because of destructive interference. The light intensities for *d* = 80 nm, 120 nm, 160 nm, and no mirror are 4.0, 1.75, 0.08, and 0.64, respectively. The important point here is that the light intensity for the no mirror condition is smaller than 1. The reason is that the emitted light from the LB film is reflected at the boundary between the LB film and air. Therefore, the maximum enhancement factor of the emission light intensity between the aluminum coated and uncoated conditions will be 6.25 (=4.0/0.64).

Figure 2b shows the FDTD simulations of the excitation beam of 405 nm for three different PMMA thicknesses. In the simulations, the p-polarized excitation beam travels at a 45° angle from air into the LB and PMMA layers and then is reflected by the aluminum layer. Note that the light intensities in the LB films are different between the three conditions. The position of the LB film is represented by a white line in Fig. 2b. The maximum light intensities in the LB film for *d* = 80 nm and no mirror are 2.0 and 0.41, respectively, while the electric field was close to zero for *d* = 160 nm. The decrease in intensity for *d* = 160 nm is due to destructive interference, and the two times increase in intensity can also be explained by interference. The reason of the two times increase is that Fig. 2b shows only the *E*_*x*_ component. The *E*_*z*_ component is also increased two times by the interference, and the electric field intensity is four times higher in total compared to the light source intensity. However, *E*_*z*_ is not shown in Fig. 2b because *E*_*z*_ is not attributed to the excitation of the LB film. As with the case of the FDTD simulation for emission, the light intensity for the no mirror condition is smaller than 1 because the excitation light is reflected at the boundary between the LB film and air. Therefore, the maximum enhancement factor of the excitation light intensity between the aluminum coated and uncoated conditions at 45° will be 4.88 (=2.0/0.41).

**Figure 3:**
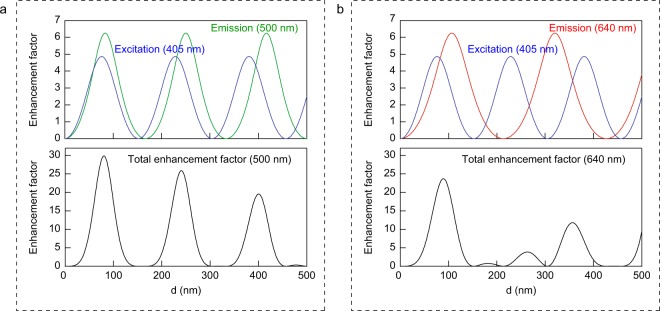
Emission, excitation, and total enhancement factors as a function of PMMA thickness *d*. Emission and excitation enhancement factors are obtained by Eq. 1: (**a**) 500 nm and (**b**) 640 nm. Total enhancements are obtained by multiplying the emission enhancements by the excitation enhancements.

To analyze the interference enhancement theoretically, the analytical solution of enhancement factor is derived based on the transfer matrix method which is widely used for analysis of a one-dimensional layered structure^45^. Since the thickness of the LB monolayer is a few nanometers, we regard the LB monolayer and the PMMA as a one layer. That is, we calculated the optical properties in a three-layered model (air/PMMA/aluminum) to obtain the enhancement factor. When the metal layer is assumed as a perfect electric conductor, the enhancement factor at the boundary between air and PMMA layer for p-polarization becomes

1 $$\frac{4{\sin }^{2}(kd\,\cos \,\theta )}{1+2{r}_{p}\,\cos (2kd\,\cos \,\theta )+{r}_{p}^{2}},$$

where *r*_*p*_ is the reflection coefficient for p-polarization at the boundary between air and PMMA, *k* is the wave number in the PMMA, and *θ* is the propagation angle of light in the PMMA. A detailed derivation of Eq. (1) is presented in the Supplementary Information. When the angle of incident for the excitation beam is 45° and the refractive index of the PMMA is 1.5, *θ* becomes 28°. This equation can adapt for the emission and excitation enhancements. Figure 3 shows the enhancement factor as a function of *d* for wavelengths of 500 nm and 640 nm. In Fig. 3a, the upper graph shows the emission and excitation enhancements, while the lower graph shows total enhancement which is obtained by multiplying the emission enhancement by the excitation enhancement. The total enhancement factor periodically changes with increasing *d*. When *d* is 80 nm, the emission, excitation, and total enhancement factors for wavelength of 500 nm are 6.19, 4.82, and 29.87, respectively. These values agree well with the FDTD simulation results of 6.25, 4.88, and 30.5. The small errors are caused by the resolution of the FDTD simulations. We consider that this good agreement provides supportive evidence for our calculations because they are independently obtained using different methods. These results show that the interference which provides approximately 30-fold enhancement can be achieved if we use a lossless perfect mirror. Figure 3b shows the enhancement factors for emission with wavelength of 640 nm. In this case, a large peak appears in the total enhancement factor curve at 89 nm, and then some weak peaks appear because of the mismatching of two emission and excitation peaks.

**Figure 4:**
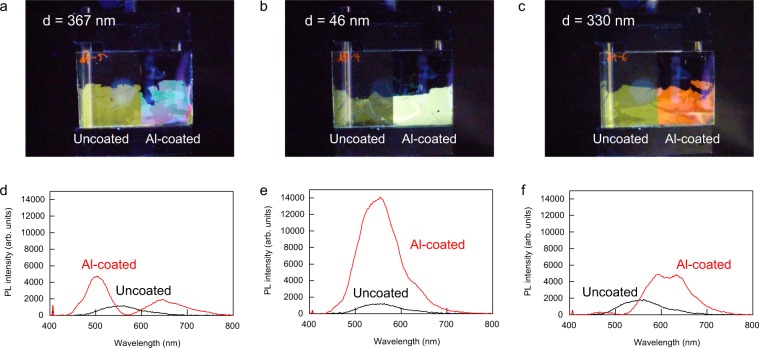
Photographs and PL spectra of the LB films on aluminum coated and uncoated regions with three different PMMA thicknesses *d*. (**a**,**d**) d = 367 nm, (**b**,**e**) d = 46 nm, and (**c**,**f**) d = 330 nm.

The thickness dependence of the enhancement factor has been experimentally confirmed. Figure 4 shows the photographs and PL spectra of the LB films with three different PMMA thicknesses. The right sides of the samples are on the aluminum coated glass substrate, while the left sides of the samples are on the non-aluminum coated glass substrate. These photographs were taken under 365 nm UV light irradiation. As is evident from Fig. 4a–c, the emission color of the LB film on the aluminum layer depends on the thickness of the PMMA spacer. The colors of the LB films on the aluminum layer with three different spacers *d* = 367 nm, 46 nm, and 330 nm are blue-green, pale yellow-green, and red, respectively. Furthermore, despite the same luminescent material, the left and right sides of all three samples show different colors. The left sides of the three samples show the same colors of dark yellow-green because there is no optical difference in the non-aluminum coated regions. The three PL spectra from the non-aluminum coated regions are almost the same, while the three PL spectra from the aluminum-coated regions are completely different. The peak wavelength of the PL emission from the LB film on the non-aluminum coated region is 550 nm. In contrast, the PL spectrum of the LB film on the aluminum layer with a 367 nm PMMA spacer has two peaks at 500 nm and 650 nm. The enhancement factors at 500 nm and 650 nm are 20-fold and 15-fold, respectively. PL intensity near the two peaks increased, but PL intensity near 570 nm is close to zero. Due to this spectral change, the color of the LB film was changed from dark yellow-green to blue-green. The PL spectra of *d* = 46 nm are shown in Fig. 4e, in which the spectral profiles of the two configurations are almost the same except intensity. Therefore, the emission color on the aluminum layer changes from dark yellow-green to pale yellow-green which is the original color of the sample. Figure 4c,f shows the photograph and PL spectra for *d* = 330 nm. As clearly seen from Fig. 4c, the color of the sample on the aluminum layer becomes red. The PL intensity above 550 nm increased and the enhancement ratio at 650 nm is 15-fold. The most interesting point here is that the emissions from the LB film not only change color but are also strongly enhanced. In general, when we use conventional optical filters to select a range of wavelengths, the light intensity always decreases because the filters selectively transmit light in a particular range of wavelengths by absorbing or reflecting light. However, the LB film on the aluminum layer shows higher intensity than the original intensity despite changing color. This is because that the color change is based on the metal enhanced emission.

**Figure 5:**
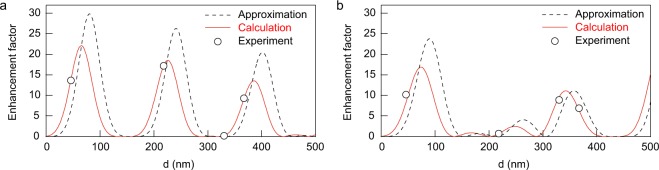
Total enhancement factors as a function of PMMA thickness *d*. Dashed curves are obtained from Eq. 1 in which metal is assumed as a perfect conductor. Red solid curves are obtained by transfer matrix method taking into account complex refractive indices of aluminum. Open circles are experimental data. Emission wavelengths of the LB films are (**a**) 500 nm and (**b**) 640 nm.

To compare between experimental and theoretical results, we have calculated the thickness dependence of the enhancement factor taking into account complex refractive indices of aluminum. The real and imaginary parts of refractive index of aluminum were taken from references^46^. In Fig. 5a, the approximation shown as the black dashed line represents the curve of the enhancement factor obtained by assuming metal as a perfect conductive layer, and the calculation shown as the red solid line represents the curve of the enhancement factor obtained by taking into account the complex refractive indices of aluminum. The dashed curve in Fig. 5a is the same as the curve in Fig. 3a. One of the differences between dashed and red solid curves in Fig. 5a,b is the peak values. The perfect conductive approximation does not include any loss component. In contrast, since the imaginary part of the refractive index corresponds to losses, the calculated curve with the complex indices shows a smaller enhancement factor than that obtained with perfect conductive approximation. The peak positions are also different between the dashed and red solid curves. This is because light slightly penetrates in the actual aluminum layer, while light cannot penetrate in the perfect conductor. On the other hand, the experimental values are plotted as open circles. The experimental results show a good agreement with the calculated curve by taking into account the complex refractive indices of aluminum. This indicates that the imaginary part of the refractive index of metal is also important for the metal enhanced emission.

**Figure 6:**
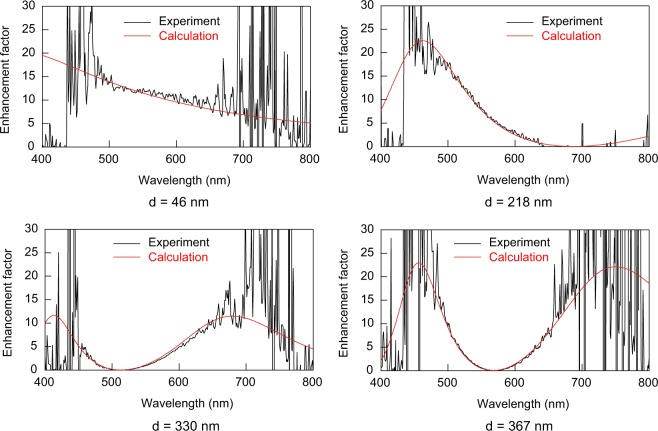
Measured and calculated enhancement factors for four different PMMA thicknesses *d*. Calculated enhancement factors are obtained by transfer matrix method taking into account complex refractive indices of aluminum.

**Figure 7:**
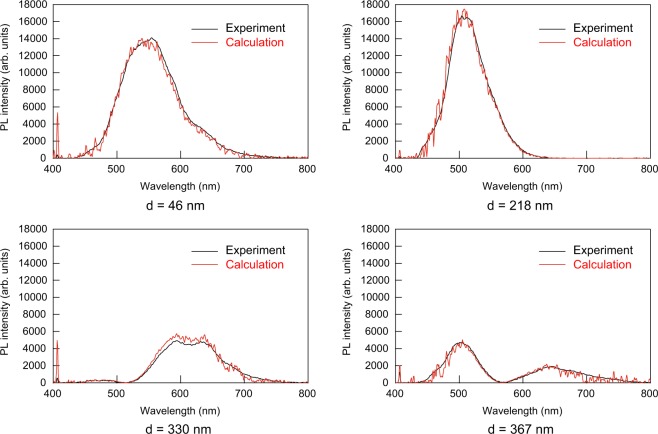
Measured and calculated emission spectra for four different PMMA thicknesses *d*. Calculated enhancement factors are obtained by multiplying the emission spectra from non-aluminum coated regions by the calculated enhancement factors shown in Fig. 6.

We also calculated enhancement factor as a function of wavelength. Figure 6 shows the measured and calculated enhancement factors for *d* = 46, 218, 330, and 367 nm. These wavelength dependences of enhancement factors were also obtained by taking into account the complex refractive indices of aluminum. At lower and higher wavelength, the experimental curves are largely affected by noise due to them being outside of the emission spectral range. However, the measured and calculated results show essential agreements in the spectral range from 450 nm to 700 nm. Furthermore, we calculated the enhanced emission spectra by multiplying the spectra obtained at the uncoated region by the enhancement factors. Figure 7 shows the measured and calculated enhanced emission spectra of LB film on the aluminum layer. The calculations can quantitatively reproduce the measured spectra.

## Discussion

Strongly-enhanced emissions from an LB film on a planar aluminum layer with various PMMA spacers have been observed. The FDTD simulations visually show us how enhance the electric fields of emission and excitation. We also derived the analytical solution as Eq. 1 using a three-layered model and the perfect conductive approximation. The large enhancements in the experiments can be explained by the interference enhancement because the measured results show good agreement with the calculations of interference enhancement. Figure 3 shows the enhancement factor due to interference as a function of PMMA thickness *d*. This indicates that we would be able to observe a 30-fold emission enhancement if we use a lossless mirror. The samples having various thicknesses of PMMA spacers show different colors depending on the PMMA thickness, as shown in Fig. 6. Although the original color of the LB film is dark yellow-green, the samples of *d* = 367 and 330 nm shows blue-green and red color, respectively. In the sample of *d* = 46 nm, emission intensity is strongly enhanced and the sample shows bright yellow-green. Conventional color filters can also change color, but generally the conventional color filters cut off a certain range of spectral signal. Therefore, the total intensity through the conventional color filters always decreases. However, the LB film on the metal layer not only changes color, but also enhances their emission intensity. Despite the very weak red spectral component in the original sample, the sample of *d* = 330 nm clearly shows a bright red color in which the red spectral component is selectively enhanced. This indicates that spectral colors of LB films can be changed without molecular design which is not easy to conduct. We believe that even the well-known interference still opens possibilities for new applications. A unique optoelectronic system using low-dimensional phase-change films have also been proposed recently, in which its color and tunability are based on interference effect^47^. Development of a new light source for biological applications is of great importance, but only a certain wavelength region can be utilized which is the so-called biological window. The interference enhancement would help to achieve the desired spectral range for such light source.

The enhancement factors calculated taking into account complex refractive indices of aluminum show a good agreement with the experimental results. The enhanced spectra could also be reproduced by the calculation. From these results, we consider that our sample does not show plasmonic enhancement. The transfer matrix calculation is very useful to analyze whether the cause of the enhancement is plasmon or interference. The enhancement factor obtained with perfect conductive approximation can be also used for rough estimation. On the other hand, if the plasmonic enhancement appears with the interference enhancement, we can obtain further enhancement. Next challenges are the implementation of simultaneous plasmonic and interference enhancements and a more precise control of emission color.

## Methods

### Preparation of LB Monolayer Film on Metal Surface

We prepared aluminum-evaporated glass substrates where half of the area of the substrate was coated with aluminum while the other half was not coated. A spacer was prepared on the glass substrate by spin coating poly(methyl methacrylate) (PMMA) 2 wt.% solution in toluene. The aluminum-evaporated glass substrate with the PMMA spacer was coated with an LB film. The PL enhancement caused by the aluminum layer was evaluated by comparing PL emission from the LB film on the aluminum-coated area with that from the LB film on the non-coated area.

LB films were prepared using an LB trough (USI System, Japan). A trough with a surface area of 10.0 cm × 12.0 cm was maintained at 20 °C by circulating water. 0.2 mL of chloroform solution containing a perchlorate salt of [Ir(dfppy)_2_(dc9bpy)]^+^ (denoted DFPPY) (ca. 5 × 10^−5^ M) was spread over an aqueous dispersion of a saponite clay (10 mg L^−1^). After 30 min, the surface was compressed at a rate of 10 cm^2^ min^−1^ until the surface pressure reached 10 mN m^−1^. Keeping the surface pressure at 10 mN m^−1^ for 30 minutes, the film was transferred onto the aluminum-coated glass substrate with a PMMA spacer by the vertical deposition method at a dipping rate of 10 mm min^−1^.

### Optical Spectroscopy

Figure 1a shows the schematic illustration of PL measurement setup. A 405 nm laser diode was used as an excitation light source. The beam diameter was 1.1 × 2.2 mm. The sample was irradiated at an angle of 45° using the excitation light with p-polarization. The PL emission from the sample was measured with a spectrometer (Bluewave, StellarNet) attached to a microscope with 10x objective lens. Microscopic spectroscopy can measure the transmission spectrum of a small area, which can reduce the experimental error caused by non-uniformity of thickness. A dichroic mirror was placed between the sample and the detector of the spectrometer to cut the excitation light.

## Electronic supplementary material

Supplementray information
