# Supplementary material for: Luminescent color control of Langmuir-Blodgett film by emission enhancement using a planar metal layer
Source: Sci Rep. 2018 Nov 20;8:17119. doi: 10.1038/s41598-018-35467-4 (PMC6244085; doi:10.1038/s41598-018-35467-4)
Supplement: Supplementary file 1 — Supplementray information [file 41598_2018_35467_MOESM1_ESM.pdf]

Supplementary Information for

Luminescent color control of Langmuir-Blodgett film by emission enhancement using a planar metal layer

Ryotaro Ozaki[1,*], Tatsuya Yamada[1], Shinji Yudate[1], Kazunori Kadowaki[1] & Hisako Sato[2]

[1]Department of Electrical and Electronic Engineering and Computer Science, Graduate School of Science and Engineering, Ehime University, Matsuyama, 790-8577, Japan
[2]Department of Chemistry, Graduate School of Science and Engineering, Ehime University, Matsuyama, 790-8577, Japan
*ozaki.ryotaro.mx@ehime-u.ac.jp

We here discuss light propagation in a three-layered structure (Air / PMMA / Aluminum) to calculate the emission enhancement factor. Figure S1 shows the three-layered model which consists of three different index materials: A, B, and C. The electric field of the LB monolayer is obtained from the boundary between A and B. In our experiment, the p-polarized beam was used to excite the LB film. When the angle of incidence is 0 degrees and the direction of propagation is parallel to the *z*-axis, the p-polarized light propagating in each layer can be written by

$$H_{ay}(z,t) = H_i \exp\{\, i(k_a z - \omega t)\} + H_r \exp\{- i(k_a z + \omega t)\} \quad (1)$$

$$H_{by}(z,t) = H_1 \exp\{\, i(k_b z - \omega t)\} + H_2 \exp\{ -i(k_b z + \omega t)\} \quad (2)$$

$$H_{cy}(z,t) = H_t \exp\{\, i(k_c z - \omega t)\} \quad (3)$$

$$E_{ax}(z,t) = \frac{k_a}{\varepsilon_a \omega}[H_i \exp\{\, i(k_a z - \omega t)\} - H_r \exp\{ -i(k_a z + \omega t)\}] \quad (4)$$

$$E_{bx}(z,t) = \frac{k_b}{\varepsilon_b \omega}[H_1 \exp\{\, i(k_b z - \omega t)\} - H_2 \exp\{ -i(k_b z + \omega t)\}] \quad (5)$$

$$E_{cx}(z,t) = \frac{k_c}{\varepsilon_c \omega} H_t \exp\{\, i(k_c z - \omega t)\} \quad (6)$$

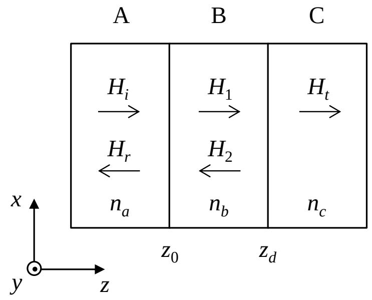

Fig. S1. Three-layered model for transfer matrix method.

,where k_a, k_b, and k_c are wavevectors, ε_a, ε_b, and ε_c are permittivitys, H_i, H_r, H_1, H_2, and H_t are amplitudes of the forward and backward propagating magnetic fields in the layers.

The boundary conditions for H_y and E_x at $z = z_0$ are given by

$$H_i \exp(ik_a z_0) + H_r \exp(-ik_a z_0) = H_1 \exp(ik_b z_0) + H_2 \exp(-ik_b z_0) \tag{7}$$

$$\frac{k_a}{\varepsilon_a} H_i \exp(ik_a z_0) - \frac{k_a}{\varepsilon_a} H_r \exp(-ik_a z_0) = \frac{k_b}{\varepsilon_b} H_1 \exp(ik_b z_0) - \frac{k_b}{\varepsilon_b} H_2 \exp(-ik_b z_0) \tag{8}$$

The two equations are based on continuity of the magnetic and electric fields at the boundary. The equations can be rewritten as a matrix form:

$$\begin{pmatrix} H_i \\ H_r \end{pmatrix} = \begin{pmatrix} \frac{\varepsilon_b k_a + \varepsilon_b k_b}{2\varepsilon_b k_a} \exp\{-i(k_a - k_b) z_0\} & -\frac{\varepsilon_b k_a - \varepsilon_b k_b}{2\varepsilon_b k_a} \exp\{-i(k_a + k_b) z_0\} \\ \frac{\varepsilon_b k_a - \varepsilon_b k_b}{2\varepsilon_b k_a} \exp\{i(k_a + k_b) z_0\} & \frac{\varepsilon_b k_a + \varepsilon_b k_b}{2\varepsilon_b k_a} \exp\{i(k_a - k_b) z_0\} \end{pmatrix} \begin{pmatrix} H_1 \\ H_2 \end{pmatrix}$$

$$= \begin{pmatrix} M_{11} & M_{12} \\ M_{21} & M_{22} \end{pmatrix} \begin{pmatrix} H_1 \\ H_2 \end{pmatrix} \tag{9}$$

When light enters from medium A into medium B with an incident angle of θ_a, M_{ij} can be written as

$$M_{11} = \frac{\varepsilon_b k_a \cos\theta_a + \varepsilon_b k_b \cos\theta_b}{2\varepsilon_b k_a \cos\theta_a} \exp\{-i(k_a \cos\theta_a - k_b \cos\theta_b) z_0\} \tag{10}$$

$$M_{12} = -\frac{\varepsilon_b k_a \cos\theta_a - \varepsilon_b k_b \cos\theta_b}{2\varepsilon_b k_a \cos\theta_a} \exp\{-i(k_a \cos\theta_a + k_b \cos\theta_b) z_0\} \tag{11}$$

$$M_{21} = \frac{\varepsilon_b k_a \cos\theta_a - \varepsilon_b k_b \cos\theta_b}{2\varepsilon_b k_a \cos\theta_a} \exp\{i(k_a \cos\theta_a + k_b \cos\theta_b) z_0\} \tag{12}$$

$$M_{22} = \frac{\varepsilon_b k_a \cos\theta_a + \varepsilon_b k_b \cos\theta_b}{2\varepsilon_b k_a \cos\theta_a} \exp\{i(k_a \cos\theta_a - k_b \cos\theta_b) z_0\} \tag{13}$$

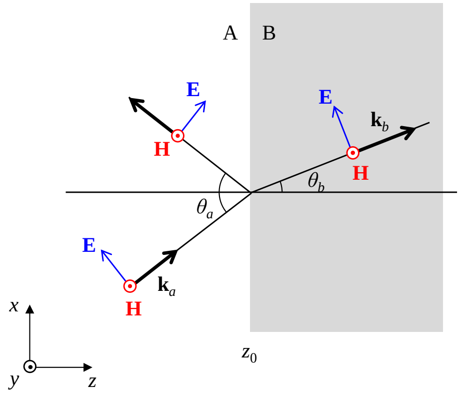

Fig. S2. Light propagation for p-polarization with an incident angle of θ_a.

Under the oblique incidence condition, the *x*-component of the electric field in the material B is given by

$$E_{bx}(z,t) = \frac{k_b \cos\theta_b}{\varepsilon_b \omega}\{H_1 \exp(ik_b z \cos\theta_b) - H_2 \exp(-ik_b z \cos\theta_b)\} \exp(-i\omega t) \qquad (14)$$

To obtain the enhancement factor between with- and without-mirror conditions, we will discuss the intensity of E_{bx} at the boundary between A and B later.

Figure 3S shows the three-layered structure including a mirror layer. Here the mirror is assumed as a perfect electric conductor. When the metal layer is a perfect electric conductor, the boundary condition for magnetic field at $z = d$ become a free end condition as

$$H_2 \exp(-ik_b d \cos\theta_b) = H_1 \exp(ik_b d \cos\theta_b)$$
$$H_2 = H_1 \exp(i2k_b d \cos\theta_b) \qquad (15)$$

On the other hand, the boundary conditions at $z = 0$ are given by

$$H_i + H_r = H_1 + H_2 \qquad (16)$$
$$\frac{k_a \cos\theta_a}{\varepsilon_a} H_i - \frac{k_a \cos\theta_a}{\varepsilon_a} H_r = \frac{k_b \cos\theta_b}{\varepsilon_b} H_1 - \frac{k_b \cos\theta_b}{\varepsilon_b} H_2 \qquad (17)$$

By adding the two equations, H_r is eliminated. And then, by substituting Eq. (15) into the equation, we obtain

$$\frac{n_b}{n_a}\left(\frac{2n_a \cos\theta_a}{n_b \cos\theta_a + n_a \cos\theta_b}\right) H_i = \left(1 + \frac{n_b \cos\theta_a - n_a \cos\theta_b}{n_b \cos\theta_a + n_a \cos\theta_b} e^{i2k_b d \cos\theta_b}\right) H_1 \qquad (18)$$

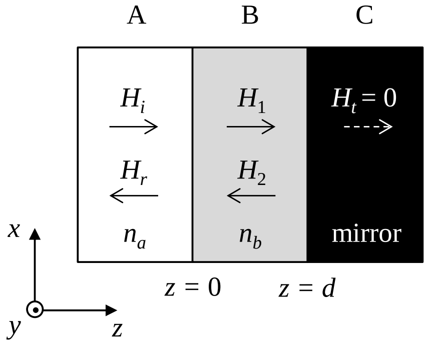

Fig. 3S. Three-layered model including a mirror layer.

The transmission coefficient t_p and the reflection coefficient r_p for p-polarization light are given by

$$t_p = \frac{2n_a \cos\theta_a}{n_b \cos\theta_a + n_a \cos\theta_b} \tag{19}$$

$$r_p = \frac{n_b \cos\theta_a - n_a \cos\theta_b}{n_b \cos\theta_a + n_a \cos\theta_b} \tag{20}$$

Using the t_p and r_p, Eq. (18) is rewritten as

$$H_1 = \frac{n_b}{n_a}\left(\frac{t_p}{1 + r_p e^{i2\alpha}}\right) H_i \tag{21}$$

,where $\alpha = k_b d\cos\theta_b$. At $z = 0$, the x-component of the electric field in the material B is given by

$$E_{bx}(0,t) = \frac{k_b \cos\theta_b}{\varepsilon_b \omega}(H_1 - H_2)\exp(-i\omega t) \tag{22}$$

Here, the amplitude E_{bx0} of this electric field can be written as

$$\begin{aligned} E_{bx0} &= \frac{k_b \cos\theta_b}{\varepsilon_b \omega}(H_1 - H_2) \\ &= \frac{\cos\theta_b}{n_a \varepsilon_0 c} \cdot \frac{t_p\left(1 - e^{i2\alpha}\right)}{1 + r_p e^{i2\alpha}} H_i \end{aligned} \tag{23}$$

Therefore, the intensity ratio between E_{bx0} and H_i can be obtained as

$$\left|\frac{E_{bx0}}{H_i}\right|^2 = \left(\frac{\cos\theta_b}{n_a \varepsilon_0 c}\right)^2 \cdot \frac{4t_p^2 \sin^2\alpha}{1 + 2r_p \cos 2\alpha + r_p^2} \tag{24}$$

Next, we will consider the amplitude E'_{bx0} at $z = 0$ without the mirror layer. If the mirror is absent and the reflection between B and C is neglected, H_2 becomes zero. In this case, the boundary conditions at $z = 0$ are given by

$$H_i + H_r = H_1 \tag{25}$$

$$\frac{k_a \cos\theta_a}{\varepsilon_a} H_i - \frac{k_a \cos\theta_a}{\varepsilon_a} H_r = \frac{k_b \cos\theta_b}{\varepsilon_b} H_1 \tag{26}$$

By adding the two equations to eliminate H_r , we obtain

$$H_1 = \frac{2\varepsilon_b k_a \cos\theta_a}{\varepsilon_b k_a \cos\theta_a + \varepsilon_a k_b \cos\theta_b} H_i \tag{27}$$

Using Eq. (27), the amplitude E'_{bx0} under no mirror condition is written as

$$E'_{bx0} = \frac{\cos\theta_b}{n_a \varepsilon_0 c} t_p H_i \tag{28}$$

The intensity ratio between E'_{bx0} and H_i can be obtained as

$$\left|\frac{E'_{bx0}}{H_i}\right|^2 = \left(\frac{\cos\theta_b}{n_a \varepsilon_0 c}\right)^2 \cdot t_p^2 \tag{29}$$

Finally, we obtain the intensity ratio between the with- and without- mirror conditions as

$$\left|\frac{E_{bx0}}{E'_{bx0}}\right|^2 = \frac{4\sin^2\alpha}{1 + 2r_p \cos 2\alpha + r_p^2} \tag{30}$$

Equation (30) is the analytical solution of the interference enhancement factor which is derived assuming the mirror layer as a perfect electric conductor. On the other hand, when the enhancement factors taking into account complex refractive indices of aluminum are calculated, the electric fields at boundary A and B can be obtained by substituted the index values into Eq. (10)-(13).
